# Molecular Heterogeneity of Glucose-6-Phosphate Dehydrogenase Deficiency in Burkina Faso: G-6-PD Betica Selma and Santamaria in People with Symptomatic Malaria in Ouagadougou

**DOI:** 10.4084/MJHID.2016.029

**Published:** 2016-06-15

**Authors:** Abdoul Karim Ouattara, Pouiré Yameogo, Birama Diarra, Dorcas Obiri-Yeboah, Albert Yonli, Tegwindé Rebeca Compaore, Serge Théophile Soubeiga, Florencia Wenkuuni Djigma, Jacques Simpore

**Affiliations:** 1 Biomolecular Research Center Pietro Annigoni (CERBA) LABIOGENE UFR/SVT, University of Ouagadougou BP 364 Ouagadougou, Burkina Faso; 2 Department of Microbiology and Immunology, University of Cape Coast, Ghana

## Abstract

The G-6-PD deficiency has an important polymorphism with genotypic variants such as 202A/376G, 376G/542T and 376G/968T known in West African populations. It would confer protection against severe forms of malaria although there are differences between the various associations in different studies. In this study we genotyped six (06) variants of the G-6-PD gene in people with symptomatic malaria in urban areas in Burkina Faso.

One hundred and eighty-two (182) patients who tested positive using rapid detection test and microscopy were included in this study. A regular PCR with the GENESPARK G6PD African kit was run followed by electrophoresis, allowing initially to genotype six SNPs (G202A, A376G, A542T, G680T, C563T and T968C). Women carrying the mutations 202A and/or 376G were further typed by real-time PCR using TaqMan probes rs1050828 and rs1050829.

In the study population the G-6-PD deficiency prevalence was 9.9%. In addition of G-6-PD A- (202A/376G) variant, 376G/542T and 376G/968T variants were also detected. Hemoglobin electrophoresis revealed that 22.5% (41/182) of the individuals had HbAC compared with2.2% with HbAS and one individual had double heterozygous HbSC. There was no correlation between the G-6-PD deficiency or haemoglobinopathies and symptomatic malaria infections in this study.

Our study confirms that the G-6-PD deficiency does not confer protection against *Plasmodium falciparum* infections. As opposed to previous genotyping studies carried out in Burkina Faso, this study shows for the first time the presence of the variant A- (376G/968C) and warrants further investigation at the national level and in specific ethnic groups.

## Introduction

The G-6-PD deficiency and haemoglobinopathies occur with high frequency in sub-Saharan Africa due to the malaria endemicity.^1^ Indeed, the maintenance of these genetic abnormalities usually asymptomatic in the homozygous state, within populations, demonstrates the selective advantage that they confer in the heterozygous state to carriers against severe malaria.^2^ S hemoglobin responsible for sickle cell disease is probably the most common serious of haemoglobinopathies in the world. Hemoglobin C is found only in parts of West Africa with the highest frequencies observed in Burkina Faso.^3^ The beta S (β^s^) form of hemoglobin is caused by a single gene mutation: a “transversion” of the β_6_ globin gene (GAG to GTG) sixth codon of the first exon that result in the substitution of glutamic acid with valine (β_6_Glu → Val). As to the β^c^ form, it is induced by gene mutation “transition” of the β_6_ globin gene (GAG to AAG) sixth codon of the first exon that resulted in the substitution of glutamic acid for lysine (β_6_Glu→Lys). Since the Haldane hypothesis, several studies have demonstrated the protective effect of hemoglobin S and C against severe malaria although certain mechanisms remain controversial.^4^–^6^

The G-6-PD deficiency is the most common inherited enzymopathy, with over 400 million carriers worldwide.^7^ It is a genetic X-linked abnormality with various clinical expressions in heterozygous female.^8^ In humans, the G-6-PD gene is located in the telomeric region of the long arm of the X chromosome in position q28. It spans about 18 kb and contains 13 exons and 12 introns, ranging in size between 12 bp and 236 bp.^9^

The G-6-PD gene is highly polymorphic with over 180 mutations described and at least 35 mutant alleles with polymorphic frequencies. These polymorphisms are relatively common in different parts of the world.^10^,^11^ Over 85% of these mutations are single nucleotide substitutions. The enzyme deficiency is asymptomatic except in cases of infections, ingestion of certain foods or oxidizing molecules.^11^ The severity of the disease depends on the genetic variant involved. G-6-PDB is the wild allele. The G-6-PDA, a non deficient variant, is the result of a substitution of adenine for guanine in position 376 of G-6-PD gene exon 5. It is faster than G6PDB electrophoretically and it does not cause haemolysis.

Most deficient variants G-6-PDA- are usually due to a second mutation on the G-6-PDA gene.^12^ The most prevalent G-6-PDA- variant in sub-Saharan Africa and the most studied is the G-6-PDA-202A/376G. However, other variants like the G-6-PDA-376G/542T, 376G/680T, 376G/968C have been reported in some African populations, including West African populations with relatively high frequencies.^13^–^15^ Also in their study in Mali, Maiga et *al.*^15^ observed an association of other G-6-PD SNPs, including rs915942 and rs915941 with asymptomatic malaria in Dogon women. The allelic heterogeneity of the G-6-PD gene suggests the need to consider a broad range of G-6-PD variants in association studies. In this study we sought for six (06) single nucleotide polymorphisms substitution involved in the G-6-PD deficiency in patients with symptomatic malaria consulting in three health centers in the city of Ouagadougou in Burkina Faso.

## Materials and Methods

### Setting and type of study

This is a prospective study in which patients regardless of gender or ethnic group were recruited in three health centers in Ouagadougou, the capital of Burkina Faso, from September 27 to November 10, 2014. Malaria transmission is hyper-endemic and seasonal during the rainy season from June to October.

### Study Population

The study involved 182 patients aged 1 to 72 years, attending Saint Camille Hospital of Ouagadougou (HOSCO), the Medical Center of Samandin and the Biomolecular Research Center Pietro ANNIGONI (CERBA) of Ouagadougou. Patients were sent to the laboratory when malaria was suspected, and underwent a Rapid Diagnostic Test using SD Bioline Malaria Ag Pf/Pan, after which positive individuals were included with their free and informed consent on condition that they are also positive microscopy.

### Sampling

The samples consisted of venous blood samples (5 ml of blood per subject adult and child by blood 3ml) in EDTA tubes. Part of the sample has been used for the realization of the Complete Blood Count (CBC), hemoglobin electrophoresis and thick blood. After that, the remainder of the sample was centrifuged at 15000 rpm for 5 minutes to separate the plasma from the pellet, aliquoted and stored at −80 ° C for molecular analysis.

### Hematologic and thick smear

Hematological parameters were determined from blood samples with EDTA, using a blood counter ABX Micros 60 (ABX Diagnostics, Montpellier, France).

Hemoglobin Genotyping was made by electrophoresis in alkaline pH on a cellulose acetate tape. Tris-glycine at pH 9.5 was used as a buffer. The cells were washed and then lysed using 1% saponin. The migration was carried out for 60 minutes at 200 V on average.

The blades of thick films were stained for 10 minutes in a solution of 10% Giemsa. The reading was performed using an optical microscope objective 100 under oil immersion and parasite density positive slides was calculated and expressed as the number of parasites/μL. In preparing the final parasite density, trophozoites were counted simultaneously with 200 leukocytes. Two independent microscopists did quality control by a repeated examination of the blades. In the case of difference of more than 5% between the results of three readings, the average of the two closest results was then retained.

### DNA extraction and genotyping of G-6-PD deficient variants

Genomic DNA was extracted from blood pellet by the standard salting-out method.^11^ The purity and the final concentration of DNA extracts were determined using the Biodrop μLITE (Isogen Life Science N.V./S.A, Temse, Belgium). All samples were initially genotyped by standard PCR. The amplification was done using the kit GENESPARK G6PD African (Immunospark, Rome, Italy) followed by electrophoresis on agarose gel 2% for six SNPs (G202A, A376G, A542T, G680T, T968C and C563T) involved in the G-6-PD deficiency.

PCR was performed in a reaction volume of 25 μL composed of 12.5 μL of Multiplex PCR smart mix (2x), 2 μL Primer Mixture (G6PD African), 8.5 μL of sterile water and 2 μL of ‘DNA fragment (50–100 ng) of each sample. Electrophoresis was performed at 100 V for 1 hour.

Female individuals with mutations in position 202 and/or 376 were further analyzed by real-time PCR using TaqMan probes respectively (Applied Biosystems, Foster City, California, USA) include: rs1050829, rs1050828.^12^

### Statistical Analyses

Data were analyzed using the software Statistical Package for Social Sciences (SPSS) 21.0 (IBM, Armonk, NY, USA) and EpiInfo™ 7. Hardy-Weinberg equilibrium was determined in women according to the method described by Carter et *al*.^16^ Pearson’s chi-square test was used for categorical variables such as age groups, parasite density groups. ANOVA was employed in the comparison of hemoglobin and hematocrit means between groups. Non-parametric tests were used to compare the geometric mean of the parasite density. The difference was significant at p <0.05.

### Ethical Considerations

The present study was approved by the Ethics Committee on Health Research of Burkina Faso (Deliberation No. 2014-9-128). Written informed consent was obtained from the adults and guardians of children.

## Results

### Demographics and Clinical Characteristics

Our study population consisted of 50.5% (92/182) of men and 49.5% (90/182) of women aged 1 to 72 years with a mean age of 17.1 ± 13.9 years. Children under 5 years old accounted for 19.2% (35/182) of the study population noted that while 46.7% (85/182) of individuals over 15 years. Socio-demographic analysis showed that 182 patients included in this study were derived from several different ethnic groups. Mossi represented the majority ethnic group with a proportion of 76.4% (139/182) of individuals whose parents were of this ethnic group (Table 1). Note that half of the group “Others” (10/20) was made up of ethnic groups from countries in the sub-region with a predominance of Nigerians (6/10). Over 43% of patients in our study had started treatment before the completion of the thick smear test. The study population was in following with Hardy-Weinberg equilibrium (p = 0.464).

### Prevalence of deficiency G-6-PD and genotypes of hemoglobin

**Figure 1:**
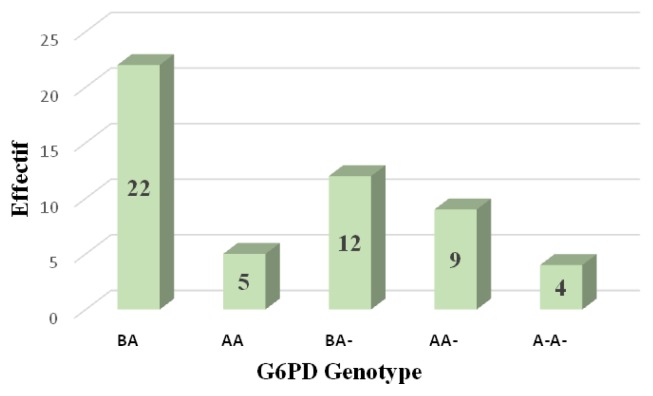
G6PD genotypes in female by real time PCR. Female homozygous = A-/A-; Female heterozygous = A/A- or B/A-; and Female normal = B/B, B/A or A/A.

Conventional PCR analysis gave more than 64% (58/90) of females carrying mutations at position 202 and/or 376. Twenty-five of them were classified as homozygous and heterozygous by real-time PCR (Figure 1). The G-6-PD deficiency genotyping showed a prevalence of 78.6% of individuals with a normal G-6-PD (29.7% genotype B, 13.2% of genotype A, 20.9% B/B, 12.1% B/A and 2.7% A/A) against 9.9% hemizygous/homozygous subjects (8.8% 202A/376G and 0.5% 376G/968C) (Table 2). Note that both parents of the individual carrier of G-6-PDA- (376G/968C) were Mossi while the parents of the female carrying the G-6-PDA- (376G/542T) variant were of the Gouro ethnic group of Ivory Coast.

**Figure 2:**
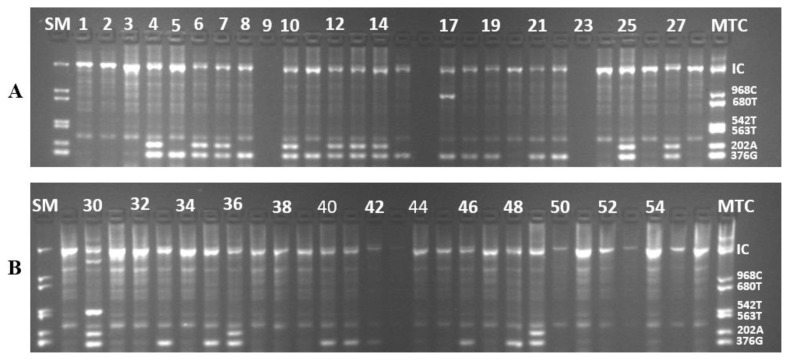
Different bands after electrophoresis(**A**) Sample number 17 carries 376G/968T mutations (**B**) Sample number 30 carries 202A/376G/542T mutations. **SM =** Standard Marker, **MTC** = Mutant Type Control, **IC** = Internal Control

She had the particularity of bearing G-6-PDA- (202A/376G) on one of the X chromosome and G-6-PDA- (376G/542T) on the other X chromosome with a parasite density of 40 parasites/μL. Figure 2 shows the bands of the different variants observed after electrophoresis.

Heterozygous females accounted for 11.5% (6.6% B/A- and 4.9% A/A-) of the study population. The prevalence of the hemizygous males was significantly higher than homozygous females (15.5% vs. 4.4% p = 0.015). The prevalence of hemoglobin genotypes was estimated to be 73.6%, 22.5% and 2.2% respectively for the genotypes AA, AC and AS. The allele frequency of HbS was 0.014 against 0.126 for HbC allele. The distribution of these genotypes was similar by sex and G-6-PD status (Table 3).

### Correlation between polymorphisms of G-6-PD genes and HBB and symptomatic malaria

The mean hemoglobin level in the study population was 11.7 g/dL ± 1.9. Approximately 66.0% (120/182) of patients had a hemoglobin level greater than 11g/dL. The majority (89.9% or 29/35) of children under the age of 5 had a hemoglobin below the level of 11 g/dL. However the distribution of hemoglobin and hematocrit was similar regardless of the G-6PD status and the hemoglobin genotype.

In the group of persons who have not started treatment prior to completion of thick smear, parasitaemia geometric mean was relatively lower in G-6PD hemizygous/homozygous individuals compared to non-deficient (3101.494 parasites/μL vs. 6879, 702 parasites/μL). Heterozygous females in this group had a similar parasite density than non-deficient individuals (7796.907 parasites/μL vs. 6879.702 parasites/μL). However, the correlation between the geometric mean of parasite density and G-6-PD status gave no statistically significant result (Table 1).

Among individuals carrying the S and/or C alleles(AS, AC, CC and SC) of hemoglobin were noted a mean parasite density of 2823.703 parasites/μL against 4454.813 parasites/μL for HbAA subjects with no statistically significant difference (p = 0.362).

## Discussion

G-6-PD has a considerable polymorphism with many genotypic variants known.^10^ The Genotyping of six (06) SNPs involved in this genetic disease has allowed us to evaluate the frequency of different deficient variants in symptomatic malaria patients. The GENESPARK G6PD African Kit (Immunospark, Rome, Italy) through a conventional PCR followed by electrophoresis without enzymatic digestion is convenient and has the advantage of having an overview of six SNPs for each sample. However, the kit is limited when it comes to genotyping women because it indicates the presence of the mutation without allowing the distinction between homozygous and heterozygous. The prevalence of hemizygous/homozygous subjects was estimated at 9.9% in our study population. This prevalence is similar to those reported by the genotyping studies in Burkina Faso and the sub-region.^12^,^16^ With respect to gender, there was a significantly higher prevalence of hemizygous male (15.5%) than homozygous female (4.4%) since the disease is X-linked.^11^ Among people with G-6-PD deficiency in this study, the G-6-PDA- (202A/376G) was the most common variant observed in 88.9% of deficiency cases. These observations are consistent with previous genotyping studies.^12^,^16^ The G-6-PD Betica Selma (376G/968C) was found in an individual of the Mossi ethnic group. This variant was observed in relatively high frequencies in the Gambian population,^14^ and in this study it is identified for the first time in Burkina Faso. A study in Mali reported a high frequency of this variant in the Fulani (6.1%) compared to the Dogon (0.0%).^15^

A deficient woman of ethnic Gouro (Ivory Coast) in our study shows the A- variants (202A/376G) and Santamaria (376G/542T) on both X chromosomes with very low parasitaemia. All these observations suggest that these variants exist in our populations even if they occur with relatively low frequency in some areas. The highest frequencies of the G-6-PD Santamaria were reported in Sere population in Senegal.^13^

Our present results show that there is an underestimation of genotypes real prevalence causing the G-6-PD deficiency in Burkina Faso. The latter could explain the different frequencies of the deficit between genotyping studies and enzymatic quantification studies in our populations.^1^,^17^

As part of this study, patients underwent a hemoglobin electrophoresis. A major sickle cell syndrome (SC) was demonstrated in 0.5% (1/182) of patients and 2.2% (4/182) had sickle cell trait AS. The most detected hemoglobin genotype was heterozygous AC present in 22.5% (41/182) of patients with 1.1% (2/182) of CC homozygosity. The prevalence of HbAC found in our study is comparable to that (19.1%) found by Simporeet*al*.^1^ in 2007 and is higher than that found by Kafando et *al.*^18^ in 2005 among newborns (15.4%). Prevalence of 14.7% and 13.0% of the AC hemoglobin were reported respectively by Amoako et *al*.^19^ in Ghana and Travassos et *al*.^20^ in Mali. All these studies show a higher rate of hemoglobin C in the sub-region. Indeed, West Africa is the epicenter of hemoglobin C.^3^

Although a reduced parasitaemia was observed in some cases (G-6-PD deficient vs.G-6-PD non-deficient individuals without treatment or HbAA subjects vs. HbS/HbC subjects), all analyses of the association between the G-6-PD status, hemoglobin genotypes, parasite density, and rate of hemoglobin or hematocrit gave no statistically significant differences. Our results are similar to those reported by Carter et *al*.^16^ in 2011 in six African countries including Burkina Faso. The authors did not observe significant effects of G-6-PD genotypes on the hemoglobin and parasitaemia. The lack of correlation between haemoglobins and G-6-PD genotypes is expected, since respective genes are on different chromosomes; and it has been reported by a previous study.^21^ The latter results also confirm that there is no protection against malaria infections. Indeed, the G-6-PD deficiency or haemoglobinopathies S and C did not offer protection against *Plasmodium falciparum* infections but may allow a favorable evolution of malaria.

The protection of the G-6-PD deficiency against severe form of malaria or malaria mortality is known, but the mechanism of protection is not entirely elucidated.^22^

In 2009 in a study in Gambia, Clark et *al*.^14^ did not find an association between severe malaria and the 202A/376Gvariant only. However, pooling this variant with other deficiency alleles revealed the signal of protection. In 2014 in Mali, Maiga et *al*.^15^ found no conclusive results on the protective effect of different G-6-PD genotypes correlated with uncomplicated malaria. The authors also suggest a higher risk of moderate malaria signs in Dogon 202A mutation carriers, especially in women. G-6-PD deficiency protective effects against cerebral malaria and an increased risk of severe malaria anemia have been reported in some studies investigating the correlation between this genetic disease and severe malaria.^23^,^24^ The allelic heterogeneity of the G-6-PD, phenotypic complexity and the difficulties of classification of clinical forms of malaria are all factors that can explain the differences between the different studies. The correlation between G-6-PD deficiency and protection against asymptomatic malaria has been reported in the literature.^12^,^25^ The selective advantage against malaria of G-6-PD heterozygous females has been early reported by Bienzle et *al*.^26^ In a case-control study carried out in Tanzania, it was established through the number of G-6-PD SNPs, that only heterozygous women were protected against severe forms of malaria.^27^ Another study in the Gambia had led to the same conclusion with variant G-6-PDA- (376G/968C).^28^ In a case-control and cohort study in Kenya, Uyoga et *al.* showed, comparing boys and girls a significant protection from severe malaria among G6PD c.202T heterozygous girls but no evidence for protection among G6PD c.202T hemizygous boys and homozygous girls (OR 1·18, 0·99-1·40; p=0·056), thus the key to protection from severe malaria could be the girls heterozygous for G6PD deficiency.^29^

Our study population size was limited in deepening the analysis. Parameters such as treatment before completion of thick smears are factors that may influence parasite density and bias the association analyses.

In 2015 in Nigeria Igbeneghu et *al*.^30^ reported a strong protection of HbAS and HbAC genotypes against asymptomatic *Plasmodium falciparum*. Protection of HbAC carriers against clinical forms of *Plasmodium falciparum* malaria has also been reported in Mali.^20^

During the same year, Mangano et *al*.^5^ reported in Burkina Faso that HbAS genotype was associated with a 70% reduction of parasite *Plasmodium falciparum* unlike HbAC carriers, although a strong protection was also observed in HbCC and HbSC subjects. Our results suggest that even if haemoglobinopathies S or C protect against severe forms of malaria like G-6-PD deficiency, they do not confer protection against *Plasmodium falciparum* infections.

## Conclusion

Our study confirms that the G-6-PDA- variant (202A/376G), the most common in Burkina Faso, does not confer protection against *Plasmodium falciparum* malaria infections. However, it shows that other variants such as T968C and probably A542T exist in our population. Further investigations are required in a larger population with well certain ethnic groups for a real estimate of the prevalence of Glucose-6-phosphate dehydrogenase variants involved in a possible association with the resistance to various kinds of malaria.

## Figures and Tables

**Table 1:** Socio-demographic and clinical Characteristics according to G-6-PD status

Characteristics	Hemizygous & homozygous (N = 18)	Heterezygous (N = 21)	Normal (N = 143)	Total (N = 182)	p
**Age (years)**					
< **5, n (%)**	4 (22.2)	0 (0.0)	31 (21.7)	35 (19.2)	
**5 à 15, n (%)**	4 (22.2)	6 (28.6)	52 (36.3)	62 (34.1)	0.051
> **15, n (%)**	10 (55.6)	15 (71.4)	60 (42.0)	85 (46.7)	
**Mean, [interval]**	17.1 [1 – 34]	26.8 [7 – 60]	15,6 [1 – 72]	17.1 [1 – 72]	0.002
**Parasitaemia (parasites/μL)**					
< **1 000, n (%)**	4 (22.2)	5 (23.8)	40 (28.0)	49 (26.9)	
**1 000 – 10 000, n (%)**	6 (33.3)	5 (23.8)	40 (28.0)	51 (28.0)	0.927
> **10 000, n (%)**	8 (44.4)	11 (52.4)	63 (44.0)	82 (45.1)	
**geometric mean μL-1 [interval]**	4235.470 [100 – 81300,0]	3750.758 [40 – 96000,0]	3945.445 [40 – 177778,0]	3950.090 [40 – 177778,0]	0.763
**Hemoglobin, mean g.dL-1**	11.8961	11.9586	11.6349	11.6981	0.704
**Hematocrit, mean %**	37.0913	38.1373	35.1266	35.6529	0.368
**Hemoglobin genotypes**					
**HbAA, parasites mean/μL**	6541.271	4511.546	4748.802	4454.813	
**HbS/C, parasites mean/μL Treatment**	2211.760	1238.458	2320.111	2823.703	0.362
**No, n (%)[Parasitaemia mean/μL]**	13 (72.2) [9522.729]	12 (57.1) [7796.907]	78 (54.5) [6879.702]	103 (56.6) [6312.948]	0.357
**Yes, n (%)[Parasitaemia mean/μL]**	5 (27.8) [3101.494]	9 (42.9) [1413.779]	65 (45.5) [2024.546]	79 (42.9) [2143.487]	0.500
Ethnic groups*					
**Mossi n (%)**	13 (72.2)	14 (66.7)	112 (78.3)	139 (76.4)	
**Mix n (%)**	4 (22.2%)	2 (9.5%)	17 (11.9%)	23 (12.6)	0.221
**Others n (%)**	1 (5.6)	5 (23.8%)	14 (9.8)	20 (11.0)	

* **Mossi**= individuals whose two parents are Mossi, **Mix** = One of the parent is Mossi, **Others** = None of the parent is Mossi.

**Table 2:** G-6-PD deficiency and its variants prevalence according to sex

G-6-PD Status	Males, n (%)	Females, n (%)	Total, N (%)
Normal	78 (84.8)	65 (72.2)	143 (78.6)
Heterozygous	0 (0.0)	21 (23.3)	21 (**11.5)**
Hemi & homozygous	14 (**15.2)**	4 (**4.4)**	18 (**9.9)**
Total	92 (100.0)	90 (100.0)	182 (100.0)
Variants A-			
202A/376G	13 (92.9)	3 (75.0)	16 (88.9)
202A/968C	1 (7.1)	0 (0.0)	1 (5.5)
202A/376G/542T	0 (0.0)	1 (0.25)	1 (5.5)
Total	14 (100.0)	4 (100.0)	18 (100.0)

**Table 3:** Study population characteristics according to HBB genotypes and HbS, HbC allele frequencies

	Individuals in functions of their hemoglobin genotypes						Frequency	
		
Factors	AA	AC	AS	CC	SC	Total	HbS	HbC
**Sex**								
**Male**	68	20	3	1	0	92	0.016	0. 120
**Female**	66	21	1	1	1	90	0.011	0.133
**G-6-PD Status**								
**Normal**	106	32	3	1	1	143	0.014	0.122
**Heterozygous**	18	3	0	0	0	21	0.000	0.071
**Hemi** & **homozygous**	10	6	1	1	0	18	0.028	0.222
**Age Groups**								
**< 5 years**	25	9	1	0	0	35	0.014	0.129
**5 – 15 years**	47	10	3	2	0	62	0.024	0.113
**> 15 years**	62	22	0	0	1	85	0.006	0.135
**Total**	134	41	4	2	1	182	0.014	0.126
